# Network analysis of anxiety and cognitive impairment among mental healthcare workers

**DOI:** 10.3389/fpsyt.2024.1393598

**Published:** 2024-08-21

**Authors:** Ruirui Chen, Wei Yan, Qinge Shen, Meng Li, Min Chen, Jicheng Dong, Yaping Wang, Xianxian Zhao, Jian Cui

**Affiliations:** ^1^ Clinical lab, Shandong Daizhuang Hospital, Jining, China; ^2^ Precision Medicine Laboratory, Shandong Daizhuang Hospital, Jining, China; ^3^ Department of Psychiatry, School of Mental Health, Jining Medical University, Jining, China; ^4^ Department of Psychiatry, Shandong Daizhuang Hospital, Jining, China; ^5^ Qingdao Mental Health Center, Qingdao, China; ^6^ The National Clinical Research Center for Mental Disorders & Beijing Key Laboratory of Mental Disorders, Beijing Anding Hospital & the Advanced Innovation Center for Human Brain Protection, Capital Medical University, Beijing, China; ^7^ Blood Transfusion Department, Jining First People’s Hospital, Shandong First Medical University, Jining, China

**Keywords:** anxiety, cognitive impairment, comorbidity, network analysis, mental healthcare workers, PDQ-D, GAD-7

## Abstract

**Introduction:**

With the rising demand for medical services and the associated burden, work-related stress and mental health issue have garnered increased attention among healthcare workers. Anxiety, cognitive impairment, and their comorbidities severely impact the physical and mental health as well as the work status of healthcare workers. The network analysis method was used to identify the anxiety and cognitive impairment among mental healthcare workers using the Generalized Anxiety Disorder Scale (GAD-7) and the Perceived Deficit Questionnaire for Depression (PDQ-D). We sought to identify the core symptoms associated with the comorbidity of anxiety and cognitive impairment in mental healthcare workers.

**Methods:**

The study was conducted by Shandong Daizhuang Hospital and Qingdao Mental Health Center in China from September 13, 2022, to October 25, 2022, involving a total of 680 healthcare workers as participants. GAD-7 and PDQ-D were utilized to assess anxiety and cognitive impairment, respectively. Regularized partial correlation network analysis was employed to examing the expected influence and predictability of each item within the network. Statistical analysis and visualization of the network were performed using R software.

**Results:**

The mean total score for anxiety was 3.25, while the mean total score for cognitive symptoms was 15.89. PDQ17 “Remembering numbers”, PDQ12 “Trouble get started” and PDQ20 “Trouble make decisions” emerged as central symptoms in the anxiety-cognition network. GAD6 “Irritable”, GAD5 “Restlessness” and GAD1 “Nervousness or anxiety” were identified as the most critical bridge symptoms connecting anxiety and cognition. Gender was found to be unrelated to the global strength of the network, edge weight distribution, or individual edge weights.

**Conclusion:**

Utilizing central and bridge symptoms (i.e., Remembering numbers, Trouble get started, Trouble make decisions, Irritable, Restlessness and Nervousness or anxiety) as primary intervention points may aid in mitigating the serious health consequences of anxiety, cognitive impairment, and comorbidities anxiety and cognitive impairment for mental healthcare workers.

## 1 Introduction

In recent years, the continuous improvement of medical services, coupled with the significant aging of the population has led to an increasing demand for medical care and an accompanying burden on the healthcare system. However, the quantity and quality of healthcare workers have not improved correspondingly, further exacerbating the work pressure of healthcare workers (1, 2). Research shows that the work stress and mental health problems of healthcare workers have become global public health problems, which seriously affect the physical and mental health and career of healthcare workers. Anxiety and cognitive impairment are common psychological problems among healthcare workers (3–5). These symptoms can seriously affect health care workers and their ability to perform their duties effectively. Understanding the impact of anxiety, cognition impairment, and their comorbidities on healthcare providers is critical for developing effective interventions and support systems to address these challenges (6, 7). A 2014 survey examining healthcare workers in China revealed that the prevalence of anxiety among this population was 22.9% (8). Additionally, a national study conducted in 2020 found that the prevalence of cognitive impairment varied between 9.7% and 23.3% (9). Anxiety and cognitive impairment of healthcare workers will not only affect their physical and mental health, but also have a negative impact on the quality of medical services and the safety and health of patients (10). Compared to anxiety or cognitive impairment alone, the occurrence of comorbidities may lead to more serious health problems, such as more severe disease, higher risk of chronic disease, and more severe dysfunction (11). Therefore, to minimize the serious health consequences of comorbidity with both, it is important that we study the specific characteristics of anxiety and cognitive impairment.

Anxiety and cognitive impairment include a series of symptoms, among which the anxiety symptoms are mainly threats, restlessness, irritability, sleep disturbance and nervousness, as well as palpitations, dry mouth and sweating. Cognitive impairment plays an important role in various mental and neurological diseases, mainly manifested as executive function, attention, memory, information processing speed and other functions, affecting people’s work and life quality (12–14). However, most studies evaluate the severity of anxiety and cognitive impairment solely through independent total scores on standardized scale, failing to capture the associations and interrelationships between individual symptoms.

Network analysis is a method employed to simulate complex systems by building a network model (a framework for conceptualizing psychological disorders) to analyze and visually display the relationship between multiple variables (15, 16). Within the context of network analysis, there is an interaction between symptoms in the symptom cluster of psychiatric disorders, where the value of each symptom subscale is a node representing the variable to be observed (for example, each symptom in the anxiety and cognitive impairment scales is a node of information). Different nodes were connected by edges, which represent the relationship between nodes (partial correlation) (17). Node centrality statistical indicators (such as strength and Expected Influence (EI)) are used to measure node characteristics and identify central influence symptoms in the network (15). The central symptom in the network model exhibits the strongest association with other symptoms, and it may activate other symptoms. This symptom could play a crucial role in the onset or maintenance of symptomatic syndromes (18). Therefore, preventive measures and interventions targeting central symptoms may be more effective. In addition, the network model provides a new way to understand the comorbidities of mental illness (19). For example, when a person has anxiety, a symptom in anxiety may increase the risk of cognitive impairment occurring, and a symptom here is seen as a bridge symptom in the network model. Bridge symptoms in the network model may play a key role in the occurrence and continuation of comorbidities of diseases, and also provide ideas for clinicians to prevent and treat comorbidities of mental diseases (20).

A network analysis of depression and anxiety comorbidity among Chinese clinicians revealed that “Impaired motor skills”, “Trouble relaxing” and “Uncontrollable worry” constitute the core symptoms of the overall depression-anxiety network. Additionally, “Irritability”, “Feeling afraid” and “Sad mood” emerged as the most critical bridging symptoms between depression and anxiety. Three symptoms—”Fatigue”, “Trouble relaxing” and “Nervousness”—exhibited the strongest and most negative correlation with quality of life. Notably, studies indicate that more than half of patients with depression experience cognitive dysfunction, which includes deficits in memory, executive function, attention, and reaction time. A substantial body of research supports the notion that cognitive dysfunction serves not only as a state marker of major depressive disorder (MDD) but also as a characteristic marker of MDD. Consequently, we aimed to explore the key symptoms associated with cognitive impairment and anxiety comorbidities for healthcare workers.

To date, no studies of comorbidity anxiety and cognitive impairment in clinicians have been published using the network model. Therefore, the purpose of this study was to investigate the network structure of anxiety and cognitive disorder comorbidity among mental healthcare workers in China, and to explore the relationship between anxiety and cognitive disorder symptoms.

## 2 Method

### 2.1 Research object

This is a cross-sectional study on the mental health status of healthcare workers, conducted by Shandong Daizhuang Hospital and Qingdao Mental Health Center in China from September 13, 2022 to October 25, 2022. We used “WJX” (a platform providing functions equivalent to Amazon Mechanical Turk, powered by www.wjx.cn) to design questionnaires and form QR codes to conduct surveys through WeChat, a smartphone-based social messaging app widely used in China (21). The inclusion criteria for participants were: 1. Age ≥18 years old; 2. Mental healthcare workers who can understand the assessment. The exclusion criteria were: 1. Mental healthcare workers with mental illnesses or those suffering from mental or physical conditions that may impair cognition; 2. Mental healthcare workers who are taking medications that could potentially affect cognitive function, including glucocorticoids, beta blockers, opioid analgesics, and central stimulants. All participants were required to sign electronic informed consent, and this study was approved by the Ethics Committee of Shandong Daizhuang Hospital (Jining Mental Health Center and the Second Affiliated Hospital of Jining Medical College) (Ethics number: 202208KS-1).

### 2.2 Measurement tools

Perceived Deficit Questionnaire for Depression (PDQ-D) was used to assess the cognitive impairment of healthcare workers. PDQ-D is a subjective assessment tool of cognitive impairment, which includes 20 items (22, 23). Each item asked how often cognitive deficits were experienced in the past week, scoring them on a scale of 0 (never), 1 (rarely), 2 (sometimes), 3 (often) and 4 (almost always). The PDQ-D score ranges from 0 to 80. The higher the PDQ-D score, the worse the subjective cognition. The method demonstrated excellent internal consistency and test-retest reliability in a study conducted with a Chinese population. The sample comprised not only individuals with major depression but also community volunteers. Consequently, this indicates that the PDQ-D can be effectively applied to the target population of mental health care workers.

The Generalized Anxiety Disorder7 (GAD-7) is commonly used to screen for generalized anxiety disorder disorders and longitudinal monitoring of treatment outcomes, asking suspected cases whether they use alcohol or drugs to reduce anxiety or stress, and screening for depression and suicide risk (22). The scale consists of seven questions on a 21-point scale, with 5–9 indicating mild anxiety, 10–14 indicating moderate anxiety, and 15–21 indicating severe anxiety.

### 2.3 Statistical methods

#### 2.3.1 Network analysis

Data analysis and visualization of anxiety and cognitive impairment networks in software R (R Core Team; version 4.3.0) (24). In network analysis, each symptom is defined as a node, the line between the two nodes is defined as an edge, the thicker the edge indicates the stronger the correlation, and the green and red edges indicate the positive and negative correlation, respectively. Sparse graph Gaussian Model (GGM) combined with graph minimum absolute contraction and selection operator (LASSO) method was used to estimate the network model. In order to reduce the number of pseudo-edges and improve the interpretable results, LASSO was used to regularize the network model (17). In addition, Extended Bayesian Information Criterion (EBIC) was used to select the optimal fitting model (15). According to previous studies, the optimal parameter of EBIC is 0.5.

##### 2.3.1.1 Central symptoms

In order to identify the most central (influential) symptoms in the network model of anxiety and cognitive impairment, we adopted an intensity centrality estimation approach. Strength centrality is a measure that reveals the strength of the connection between a particular node and other nodes by calculating the sum of the absolute edge weights of that node. For this analysis, we used the *bootnet* (17) and *qgraph* (25) in R package.

In addition, we estimate the predictability index of each node (the likelihood that the state of a given node can be explained by the state of its neighboring nodes) (26–28). This estimate is based on the *mgm* (26, 27) network model in the R package.

Bridge strengths in network models are widely used to identify symptoms that can connect two or more different diseases, such as anxiety and cognitive impairment. Therefore, we also estimated the bridge strength of each node, using the *networktools* of the R package in this process.

##### 2.3.1.2 Stability and accuracy of the network

The *bootnet* package is used to test the stability and accuracy of the network. First, the accuracy is evaluated by calculating the edge weight 95% confidence interval (bootstrapped samples = 1000). The narrower the confidence interval, the higher the accuracy (29). Secondly, in order to evaluate the stability of the expected influence, the correlation stability coefficient (CS-C) was calculated through the sample drop bootstrap method (subletting bootstrap=1000). CS-C represents the maximum proportion of samples that can be deleted so that the correlation between the original centrality index can reach at least 0.7 with a 95% probability. According to previous studies, the correlation coefficient should be higher than 0.5 and not lower than 0.25 (17). In order to evaluate whether the difference between the two edge weights or the strength centrality of two nodes was statistically significant, the difference test between the edge weights and the strength centrality of the nodes was conducted (1000 times bootstrap) (significance level α=0.05), and *P* < 0.05 was considered as statistically significant.

##### 2.3.1.3 Gender network comparison

The differences between the two networks (male vs female) were evaluated using the Network Comparison Test (NCT) package, this package based on permutation tests using 1000 permutation pairs subsamples (male vs female). The overall network strength (sum of absolute values of all edge weights) and network structure (distribution of edge weights) between the two networks were evaluated. In addition, the Holm-Bonferroni method of multiple comparisons was used to compare the edge invariance (30). These tests were performed with the R-package ‘NetworkComparisonTest’ version 2.2.1.

## 3 Result

### 3.1 Sample characteristics of comorbidity of anxiety and cognitive impairment

A total of 680 participants were invited to participate in this study, 658 met the study enrollment criteria and completed the assessment, including 462 (70.2%) women and 196 (29.8%) men. 484 (73.6%) with college education or above, and 494 (75.1%) married (**Supplementary Table 1**).

### 3.2 Network structure

The mean total score of anxiety was 3.25 and the mean total score of cognitive impairment was 15.89. No item was lower than 0.25 SD compared to the average amount of information (i.e., SD) of GAD-7 (M*_SD_* = 0.66 ± 0.19) or PDQ-D (M*_SD_* = 0.83 ± 0.04). In addition, the item redundancy analysis did not find any GAD-7 or PDQ-D items in the measurement redundant with other items.

**Figure 1:**
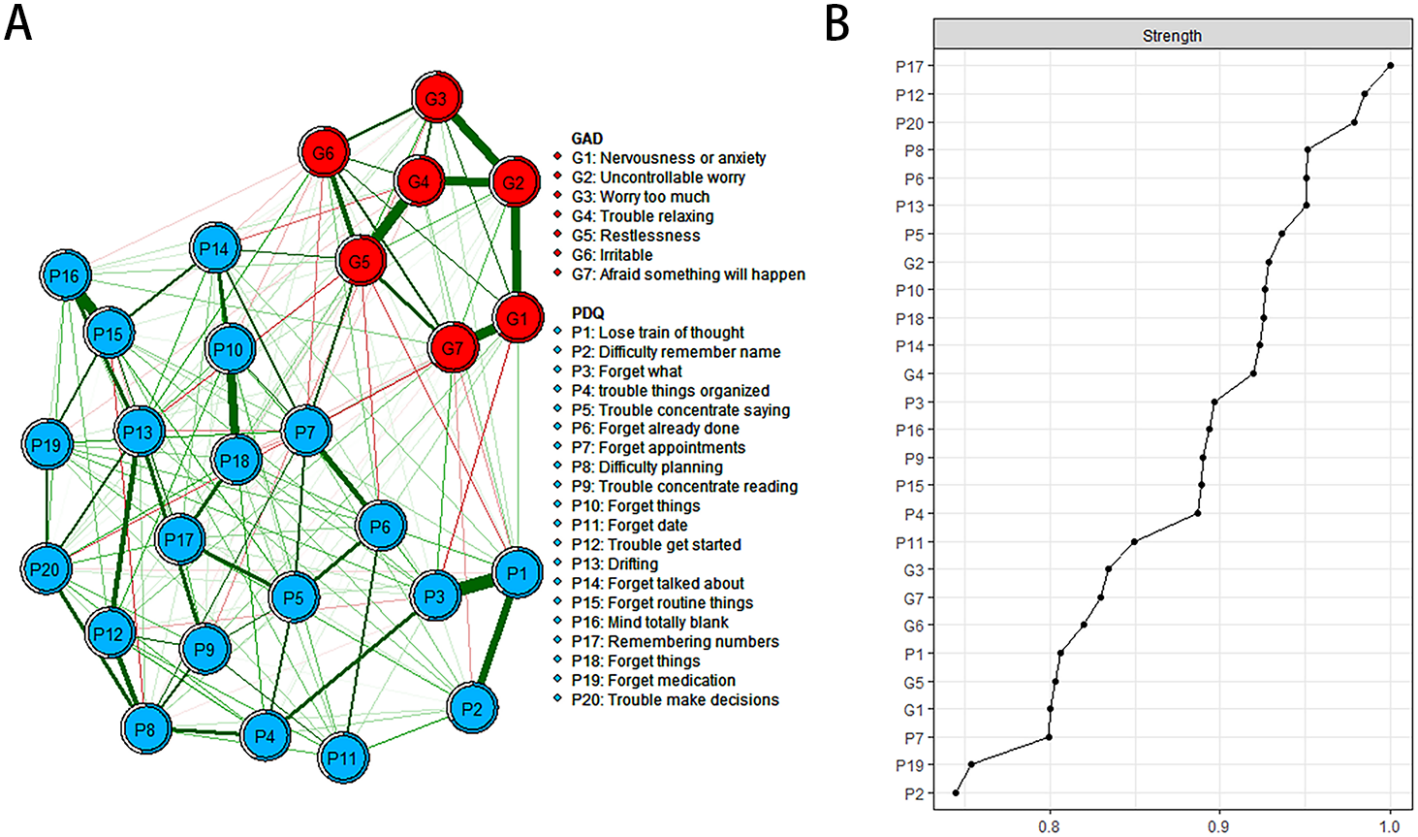
Estimated network structure of anxiety and cognitive impairment and the corresponding centrality of each node. **(A)** The anxiety and cognitive impairment network structure. The different-size circles represent different strength of the nodes, while the width and saturation of edges indicate the connections and directions (i.e., green: positive correlation; red: negative correlation). The ring around each node indicates the predictability, with a filled ring representing that 100% of the variance is accounted for by the other nodes and an empty ring corresponding to 0% predictability. **(B)** The corresponding centrality of each node in the anxiety and cognitive impairment network. PDQ-D=Perceived Deficit Questionnaire for Depression, GAD=Generalized Anxiety Disorder.

The descriptive information of cognitive deficits and anxiety symptoms is shown in **Table 1**, and the estimated network structure is shown in **Figure 1**. Several nodes are highly connected to the rest of the network, with PDQ17 “Remembering numbers” showing the highest intensity, followed by PDQ12 “Trouble get started” and PDQ20 “Trouble make decisions”.

**Table 1:** Edge weights and predictabilities of GAD-7 and PDQ-D items (n=658).

Item ID	Item Content	M	SD	Skew	Kurtosis	strength	pred
GAD1	Nervousness or anxiety	0.62	0.67	0.98	1.25	0.81	0.64
GAD2	Uncontrollable worry	0.41	0.65	1.74	3.19	1.02	0.56
GAD3	Worry too much	0.55	0.71	1.36	1.96	0.91	0.59
GAD4	Trouble relaxing	0.49	0.72	1.57	2.38	1.01	0.56
GAD5	Restlessness	0.30	0.59	2.24	5.50	0.88	0.60
GAD6	Irritable	0.55	0.68	1.17	1.48	0.90	0.60
GAD7	Afraid something willhappen	0.34	0.60	2.02	4.68	0.91	0.62
PDQ1	Lose train of thought	1.04	0.83	0.82	1.04	0.88	0.61
PDQ2	Difficulty remember name	0.99	0.91	0.82	0.38	0.82	0.66
PDQ3	Forget what	0.95	0.83	0.76	0.54	0.98	0.55
PDQ4	Trouble things organized	0.76	0.83	1.16	1.56	0.97	0.56
PDQ5	Trouble concentrate saying	0.73	0.78	1.03	1.32	1.03	0.52
PDQ6	Forget already done	0.89	0.80	0.76	0.68	1.04	0.53
PDQ7	Forget appointments	0.57	0.75	1.44	2.56	0.81	0.57
PDQ8	Forget appointments	0.72	0.84	1.26	1.68	1.04	0.53
PDQ9	Forget appointments	0.86	0.86	0.92	0.71	0.98	0.55
PDQ10	Forget things	0.67	0.79	1.24	1.86	1.02	0.51
PDQ11	Forget date	0.94	0.91	1.00	0.88	0.93	0.61
PDQ12	Trouble get started	0.74	0.86	1.35	2.20	1.08	0.51
PDQ13	Drifting	0.93	0.86	1.00	1.42	1.04	0.51
PDQ14	Forget talked about	0.62	0.76	1.23	1.74	1.01	0.54
PDQ15	Forget talked about	0.70	0.80	1.28	2.17	0.97	0.56
PDQ16	Mind totally blank	0.68	0.83	1.39	2.27	0.98	0.53
PDQ17	Remembering numbers	0.79	0.85	1.04	1.03	1.10	0.52
PDQ18	Forget things	0.85	0.88	1.03	0.99	1.01	0.53
PDQ19	Forget things	0.66	0.82	1.35	1.98	0.83	0.62
PDQ20	Trouble make decisions	0.82	0.90	1.27	1.76	1.07	0.55

M, mean; Min, minimum; Max, maximum; GAD, Generalized Anxiety Disorder; PDQ, Perceived Deficit. Questionnaire for Depression: Skew Skewness; Pred Predictability.

**Figure 2:**
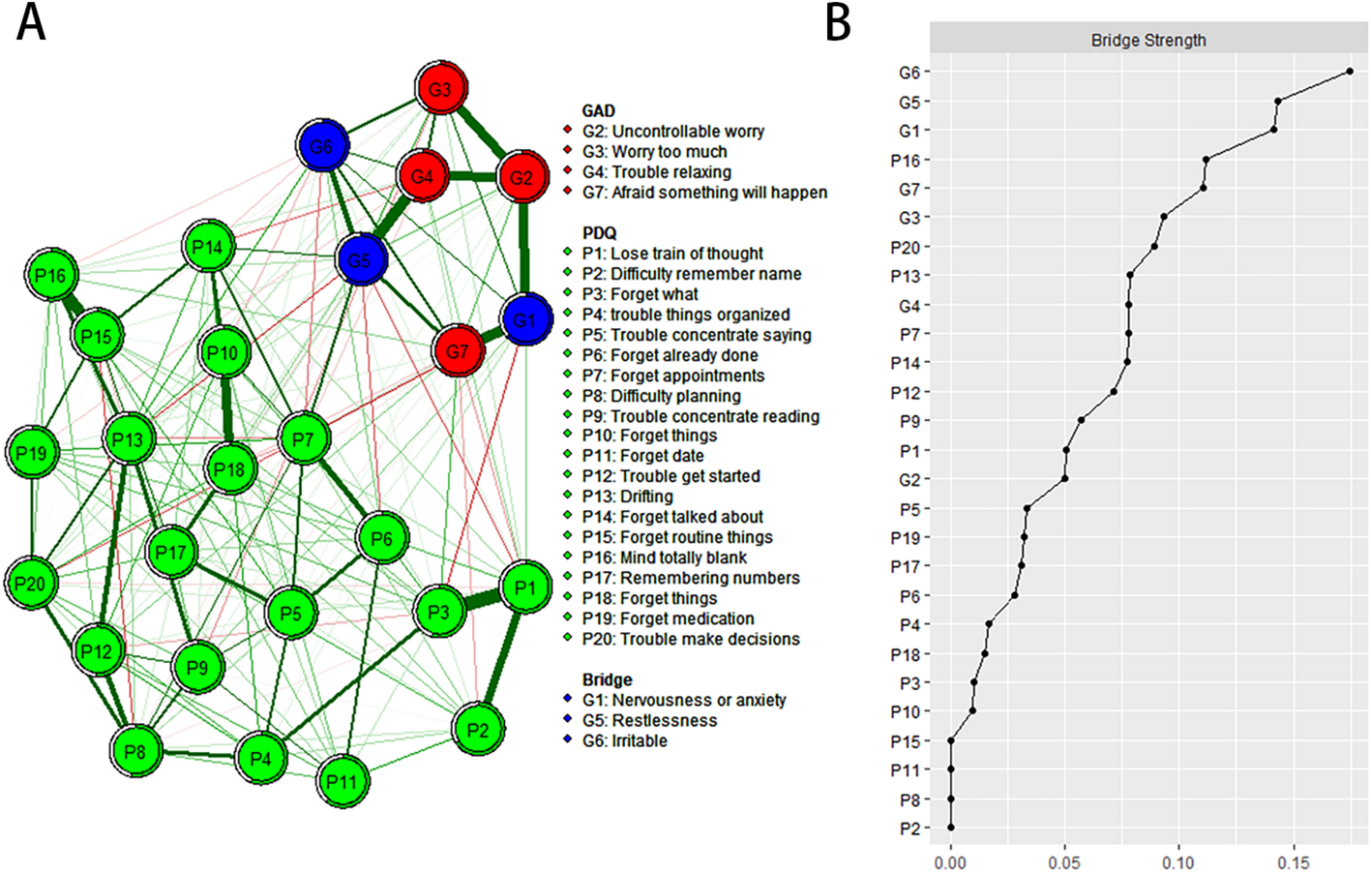
Estimated network structure of anxiety and cognitive impairment and the corresponding bridge symptoms. **(A)** The anxiety and cognitive impairment network structure highlighting the bridge symptoms. The different-size circles represent different strength of the nodes, while the width and saturation of edges indicate the connections and directions (i.e., green: positive correlation; red: negative correlation). The ring around each node indicates the predictability, with a filled ring representing that 100% of the variance is accounted for by the other nodes and an empty ring corresponding to 0% predictability. **(B)** The bridge strength of symptoms in the anxiety and cognitive impairment network. PDQ-D=Perceived Deficit Questionnaire for Depression, GAD=Generalized Anxiety Disorder.

In the anxiety cognitive network model, PDQ17 “Remembering numbers”, PDQ12 “Trouble get started” and PDQ20 “Trouble make decisions” have the highest intensity centrality. GAD6 “Irritable”, GAD5 “Restlessness” and GAD1 “Nervousness or anxiety” showed the highest bridge strength, linking cognitive deficits and anxiety symptoms in **Figure 2** and **Supplementary Table 2**.

### 3.3 Accuracy and stability of the network

**Figure 3:**
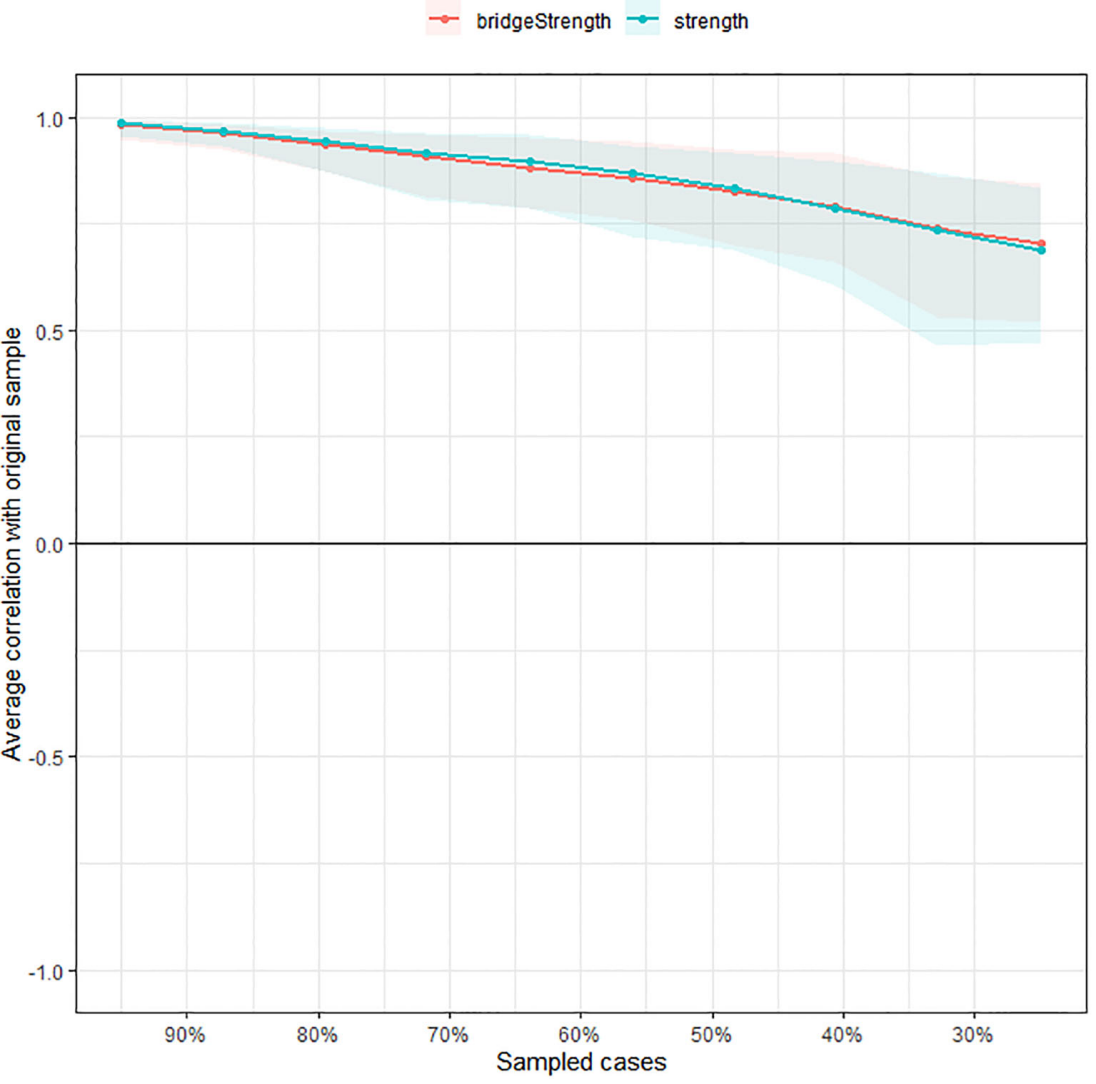
Stability of the estimated network structure of anxiety and cognitive impairment symptoms. The percentage of cases of the original sample used at each step is represented on the x-axis. The average of correlations between the centrality indices from the original network and the centrality indices from the networks that were re-estimated after excluding increasing percentages of cases is represented on the y-axis. Each line indicates the correlations among betweenness, closeness, and strength.

The accuracy of edge weights calculated by bootstrap method shows that the 95% confidence interval with significant edge weights in the current sample is narrow, indicating that the current network has good stability, as shown in **Figure 3**. The centrality of strength was calculated by subletting bootstrap=1000, and the values of edge strength and strength remained stable even after most samples were dropped (**Supplementary Figure 1**, CS-C=0.52, i.e., after dropping up to 95% of the sample, the order of the symptoms in strength was still correlated with the original one (r = 0.7)). In addition, the edge and strength difference test based on bootstrap method shows that the strongest edges and nodes are significantly different from other edges and nodes, also reflecting the high precision of the estimated network.

### 3.4 Gender network comparison

Comparing the network model between male (n = 196) and female (n = 462) healthcare workers, the overall network strength was not statistically significant (male: 11.82; female: 12.67; S=0.85, *P*=0.62; **Supplementary Figure 2**), the network difference test had no statistical significance (M =0.29, *P*= 0.73, **Supplementary Figure 3**).

## 4 Discussion

To the best of our knowledge, this study represents the first investigation in China to elucidate the network structure connecting anxiety and cognitive impairment among mental healthcare workers. In terms of network analysis, PDQ17 “Remembering numbers”, PDQ12 “Trouble get started” and PDQ20 “Trouble make decisions” are the most influential central symptoms in the network model. In addition, GAD6 “Irritable”, GAD5 “Restlessness” and GAD1 “Nervousness or anxiety” showed the highest bridge strength linking cognitive impairment and anxiety symptoms, suggesting that these symptoms should be treated first to reduce and prevent comorbidities of anxiety and cognitive impairment.

We found that PDQ17 “Remembering numbers”, PDQ12 “Trouble get started” and PDQ20 “Trouble make decisions” were central symptoms of the network model of anxiety and cognitive impairment. These symptoms correspond to the entries in PDQ-D, which provides a subjective measure of cognitive dysfunction. A study examining cognitive screening among healthcare workers over the age of 70 revealed an incidence of cognitive impairment of 14.4%. As individuals age, the risk of stroke, neurodegenerative diseases such as Alzheimer’s disease (AD), and other systemic conditions such as cancer increases, all of which can contribute to cognitive decline and elevate the risk of medical errors. This is consistent with previous research (31–36). One study found that “depression,” “anxiety” and “apathy” are common in mild cognitive impairment (MCI). These symptoms can exacerbate cognitive decline and progress to dementia (31). Data show that in patients with AD, the prevalence rate of anxiety is 9.4%-39%, and the core symptom of AD is cognitive impairment and other cognitive deficits (32–36). Other studies, however, have found something different (37–39). For example, studies focusing on cognitive features of attention-deficit/hyperactivity disorder (ADHD) will integrate “the processing speed index” (PSI) and “working memory index” (WMI). It was identified as the most serious symptom of cognitive impairment (37, 38). In a study of 50 patients with Parkinson’s disease, 33 had no anxiety and 17 had anxiety, with the latter’s core cognitive impairment being a decline in “working memory” (39). These inconsistent findings suggest differences in the central symptoms of anxiety and cognitive impairment between different study samples. In addition, the heavy workload and increased workload of healthcare workers persist (40). All these factors may increase the risk of psychomotor emergence, such as “Remembering numbers,” “Trouble get started,” and “Trouble make decisions”. In network theory, central symptoms play an important role in maintaining psychopathological networks (41), so attention and treatment of these symptoms may help improve other symptoms.

In the anxiety and cognitive impairment network model, “Irritable”, “Restlessness”, “Nervousness or anxiety” constituted the most important symptom core connecting the anxiety and cognitive disorder communities. Our study is consistent with many previous studies (42–47). For example, Roberto et al. found that irritable and apathetic patients had similar cognitive decline performance in the two memory domains, and “irritable” proved to be the most serious and fastest neuropsychiatric symptom of cognitive decline in their study (42). Studies have found that the prevalence of anxiety in patients with mild cognitive impairment or dementia ranges from 8% to 71% (43–45). “Restlessness”, “irritability”, “muscle tension”, “fear”, and “respiratory” and other anxiety symptoms reduce their cognitive and behavioral ability and their ability to live independently, resulting in a decline in their quality of life (46, 47). However, previous studies have also had inconsistent findings (48, 49). For example, in a study of 14,578 front-line healthcare workers, “Not being able to stop or control worrying” might be the core symptom and a potential effective intervention target (48). Sun et al. found that “Irritability”, “Nervousness-Uncontrollable worry” and “Excessive worry” were the central symptoms of depression and anxiety network model, and “Sad Mood”, “restlessness” and “Irritability” were the bridge symptoms of depression and anxiety comorbidities (49). We did not find more studies on the comorbidity of anxiety and cognitive impairment, suggesting that we need to fill in the gaps in the field. In conclusion, in network analysis theory, the improvement of bridge symptoms also brings changes in other symptoms of the disease (50). Therefore, interventions targeting these bridging symptoms could reduce symptoms of anxiety and cognitive impairment in healthcare workers.

This study revealed a significant role of anxiety symptoms within the network structure of cognitive impairment and anxiety comorbidity. Notably, both the central and bridge symptoms were predominantly associated with anxiety symptoms, suggesting that these symptoms play a critical role in both the onset and persistence of psychological disorders among healthcare workers. The findings support previous research. A survey of 1,685 healthcare workers revealed that 31% experienced mild anxiety, 33% exhibited clinically significant anxiety, 29% reported mild depression, and 17% faced moderate to severe depressive symptoms. The reason for this phenomenon may be related to fear and uncertainty caused by multiple factors (51). Taken together, our findings suggest the need for monitoring and intervention for specific anxiety symptoms among health care workers.

The strengths of this study include its highly representative sample and the application of network analysis methods for examining the relationship between anxiety and cognitive impairment among healthcare personnel. However, the study also has several limitations. Firstly, as a cross-sectional study, it cannot establish causal relationships between anxiety and cognitive impairment. A longitudinal study would be beneficial for gaining a deeper understanding of the subjects examined. Secondly, the assessment of anxiety and cognitive impairment relies on self-reported scales, which may limit the accuracy of clinical phenomena and introduce recall bias (52). as a cross-sectional study, it cannot establish causal relationships between anxiety and cognitive impairment. A longitudinal study would be beneficial for gaining a deeper understanding of the subjects examined. Secondly, the assessment of anxiety and cognitive impairment relies on self-reported scales, which may limit the accuracy of clinical phenomena and introduce recall bias.

In summary, the central symptoms identified by the network model of anxiety and cognitive impairment include “Remembering numbers,” “Trouble get started,” and “Trouble make decisions”. Additionally, the bridging symptoms comprise “Irritable,” “Restlessness,” and “Nervousness or anxiety”. We fill in the gaps in previous research and help identify the most impactful symptoms of anxiety and cognitive impairment comorbidities. These findings could help develop more effective interventions and prevention strategies for mental healthcare workers.

## Data Availability

The original contributions presented in the study are included in the article/**Supplementary Material**. Further inquiries can be directed to the corresponding author.
