# Supplementary material for: Network analysis of anxiety and cognitive impairment among mental healthcare workers
Source: Front Psychiatry. 2024 Aug 21;15:1393598. doi: 10.3389/fpsyt.2024.1393598 (PMC11371607; doi:10.3389/fpsyt.2024.1393598)
Supplement: Supplementary file 1 [file Table1.docx]

**Supplementary Information**

**Supplementary figure and table legends**

Table S1. Study the sociodemographic characteristics of a population(*N*=658)

Table S2. Correlation matrix of the PDQ-D and GAD-7 items

Figure S1. Nonparametric bootstrapped accuracy test for edge-weights of PDQ-D and GAD-7 items

Figure S2. Estimated network models for anxiety and cognitive impairment symptoms in male and female participants

Figure S3. Statistical difference map of global strength and maximum of difference

Table S1. Study the sociodemographic characteristics of a population(*N*=658)

| Variables | N | % |
| --- | --- | --- |
| Gender |  |  |
| Men | 196 | 29.8 |
| Women | 462 | 70.2 |
| Married/cohabiting | 494 | 75.1 |
| Education level |  |  |
| High school | 8 | 1.2 |
| Junior college | 105 | 15.9 |
| Postgraduate or higher | 379 | 57.5 |
| Low income | 211 | 25.2 |
| Age(years) | 260(31-40) | 39.5 |

Low income: monthly income < 5,000RMB

Table S2. Correlation matrix of the PDQ-D and GAD-7 items

|  | **GAD-1** | **GAD-2** | **GAD-3** | **GAD-4** | **GAD-5** | **GAD-6** | **GAD-7** | **PDQ-1** | **PDQ-2** | **PDQ-3** | **PDQ-4** | **PDQ-5** | **PDQ-6** | **PDQ-7** | **PDQ-8** | **PDQ-9** | **PDQ-10** | **PDQ- 11** | **PDQ- 12** | **PDQ- 13** | **PDQ- 14** | **PDQ- 15** | **PDQ- 16** | **PDQ- 17** | **PDQ- 18** | **PDQ- 19** | **PDQ- 20** |
| --- | --- | --- | --- | --- | --- | --- | --- | --- | --- | --- | --- | --- | --- | --- | --- | --- | --- | --- | --- | --- | --- | --- | --- | --- | --- | --- | --- |
| **GAD-1** | 0.000 |  |  |  |  |  |  |  |  |  |  |  |  |  |  |  |  |  |  |  |  |  |  |  |  |  |  |
| **GAD-2** | 0.171 | 0.000 |  |  |  |  |  |  |  |  |  |  |  |  |  |  |  |  |  |  |  |  |  |  |  |  |  |
| **GAD-3** | 0.208 | 0.173 | 0.000 |  |  |  |  |  |  |  |  |  |  |  |  |  |  |  |  |  |  |  |  |  |  |  |  |
| **GAD-4** | 0.079 | 0.252 | 0.150 | 0.000 |  |  |  |  |  |  |  |  |  |  |  |  |  |  |  |  |  |  |  |  |  |  |  |
| **GAD-5** | 0.000 | 0.147 | 0.066 | 0.233 | 0.000 |  |  |  |  |  |  |  |  |  |  |  |  |  |  |  |  |  |  |  |  |  |  |
| **GAD-6** | 0.134 | 0.037 | 0.188 | 0.149 | 0.072 | 0.000 |  |  |  |  |  |  |  |  |  |  |  |  |  |  |  |  |  |  |  |  |  |
| **GAD-7** | 0.144 | 0.187 | 0.036 | 0.067 | 0.219 | 0.144 | 0.000 |  |  |  |  |  |  |  |  |  |  |  |  |  |  |  |  |  |  |  |  |
| **PDQ-1** | 0.000 | 0.000 | 0.031 | 0.013 | 0.000 | 0.007 | 0.000 | 0.000 |  |  |  |  |  |  |  |  |  |  |  |  |  |  |  |  |  |  |  |
| **PDQ-2** | 0.000 | 0.000 | 0.000 | 0.000 | 0.000 | 0.000 | 0.000 | 0.216 | 0.000 |  |  |  |  |  |  |  |  |  |  |  |  |  |  |  |  |  |  |
| **PDQ-3** | 0.000 | 0.000 | 0.000 | 0.000 | 0.000 | 0.010 | 0.000 | 0.304 | 0.066 | 0.000 |  |  |  |  |  |  |  |  |  |  |  |  |  |  |  |  |  |
| **PDQ-4** | 0.000 | 0.000 | 0.017 | 0.000 | 0.000 | 0.000 | 0.000 | 0.017 | 0.078 | 0.146 | 0.000 |  |  |  |  |  |  |  |  |  |  |  |  |  |  |  |  |
| **PDQ-5** | 0.000 | 0.020 | 0.000 | 0.000 | 0.006 | 0.007 | 0.000 | 0.040 | 0.000 | 0.000 | 0.101 | 0.000 |  |  |  |  |  |  |  |  |  |  |  |  |  |  |  |
| **PDQ-6** | 0.011 | 0.000 | 0.000 | 0.000 | 0.000 | 0.017 | 0.000 | 0.118 | 0.032 | 0.072 | 0.048 | 0.147 | 0.000 |  |  |  |  |  |  |  |  |  |  |  |  |  |  |
| **PDQ-7** | -0.034 | 0.000 | 0.000 | 0.000 | 0.044 | 0.000 | 0.000 | 0.000 | 0.030 | 0.000 | 0.000 | 0.134 | 0.080 | 0.000 |  |  |  |  |  |  |  |  |  |  |  |  |  |
| **PDQ-8** | 0.000 | 0.000 | 0.000 | 0.000 | 0.000 | 0.000 | 0.000 | 0.000 | 0.018 | 0.000 | 0.194 | 0.084 | 0.031 | 0.144 | 0.000 |  |  |  |  |  |  |  |  |  |  |  |  |
| **PDQ-9** | 0.023 | 0.000 | 0.000 | 0.000 | 0.000 | 0.034 | 0.000 | 0.000 | 0.000 | 0.000 | 0.022 | 0.073 | 0.031 | 0.000 | 0.146 | 0.000 |  |  |  |  |  |  |  |  |  |  |  |
| **PDQ-10** | 0.000 | 0.010 | 0.000 | 0.000 | 0.000 | 0.000 | 0.000 | 0.000 | 0.000 | 0.000 | 0.000 | 0.068 | 0.099 | 0.080 | 0.053 | 0.000 | 0.000 |  |  |  |  |  |  |  |  |  |  |
| **PDQ-11** | 0.000 | 0.000 | 0.000 | 0.000 | 0.000 | 0.000 | 0.000 | 0.024 | 0.136 | 0.055 | 0.000 | 0.000 | 0.138 | 0.002 | 0.050 | 0.115 | 0.088 | 0.000 |  |  |  |  |  |  |  |  |  |
| **PDQ-12** | 0.027 | 0.003 | 0.000 | 0.000 | 0.000 | 0.000 | 0.042 | 0.000 | 0.000 | 0.000 | 0.113 | 0.038 | 0.005 | 0.024 | 0.174 | 0.148 | 0.088 | 0.093 | 0.000 |  |  |  |  |  |  |  |  |
| **PDQ-13** | 0.009 | 0.001 | 0.011 | 0.000 | -0.002 | 0.055 | 0.000 | 0.059 | 0.053 | 0.048 | 0.016 | 0.046 | 0.006 | 0.000 | 0.045 | 0.187 | 0.049 | 0.009 | 0.125 | 0.000 |  |  |  |  |  |  |  |
| **PDQ-14** | 0.000 | 0.000 | 0.034 | 0.000 | 0.043 | 0.000 | 0.000 | 0.025 | 0.009 | 0.006 | 0.094 | 0.000 | 0.000 | 0.114 | 0.013 | 0.008 | 0.214 | 0.000 | 0.037 | 0.055 | 0.000 |  |  |  |  |  |  |
| **PDQ-15** | 0.000 | 0.000 | 0.000 | 0.000 | 0.000 | 0.000 | 0.000 | 0.023 | 0.031 | 0.072 | 0.000 | 0.000 | 0.000 | 0.078 | 0.000 | 0.000 | 0.022 | 0.056 | 0.021 | 0.000 | 0.138 | 0.000 |  |  |  |  |  |
| **PDQ-16** | 0.000 | 0.004 | 0.000 | 0.037 | 0.027 | 0.000 | 0.045 | 0.000 | 0.000 | 0.000 | 0.000 | 0.109 | 0.000 | 0.000 | 0.000 | 0.032 | 0.036 | 0.000 | 0.088 | 0.094 | 0.085 | 0.212 | 0.000 |  |  |  |  |
| **PDQ-17** | 0.000 | 0.004 | 0.000 | 0.000 | 0.014 | 0.013 | 0.000 | 0.000 | 0.079 | 0.043 | 0.022 | 0.132 | 0.035 | 0.000 | 0.000 | 0.089 | 0.000 | 0.000 | 0.000 | 0.000 | 0.035 | 0.170 | 0.084 | 0.000 |  |  |  |
| **PDQ-18** | 0.000 | 0.000 | 0.000 | 0.015 | 0.000 | 0.000 | 0.000 | 0.008 | 0.056 | 0.082 | 0.000 | 0.000 | 0.096 | 0.000 | 0.000 | 0.000 | 0.209 | 0.092 | 0.000 | 0.009 | 0.099 | 0.000 | 0.000 | 0.191 | 0.000 |  |  |
| **PDQ-19** | 0.000 | 0.000 | 0.000 | 0.000 | 0.008 | 0.000 | 0.024 | 0.000 | 0.014 | 0.052 | 0.000 | 0.000 | 0.076 | 0.113 | 0.000 | 0.000 | 0.000 | 0.053 | 0.000 | 0.000 | 0.003 | 0.140 | 0.050 | 0.068 | 0.074 | 0.000 |  |
| **PDQ-20** | 0.037 | 0.008 | 0.000 | 0.013 | 0.000 | 0.031 | 0.000 | 0.000 | 0.000 | 0.027 | 0.104 | 0.021 | 0.000 | 0.000 | 0.090 | 0.066 | 0.000 | 0.021 | 0.055 | 0.160 | 0.000 | 0.012 | 0.078 | 0.116 | 0.083 | 0.151 | 0.000 |

PDQ-D=Perceived Deficit Questionnaire for Depression, GAD=Generalized Anxiety Disorder.


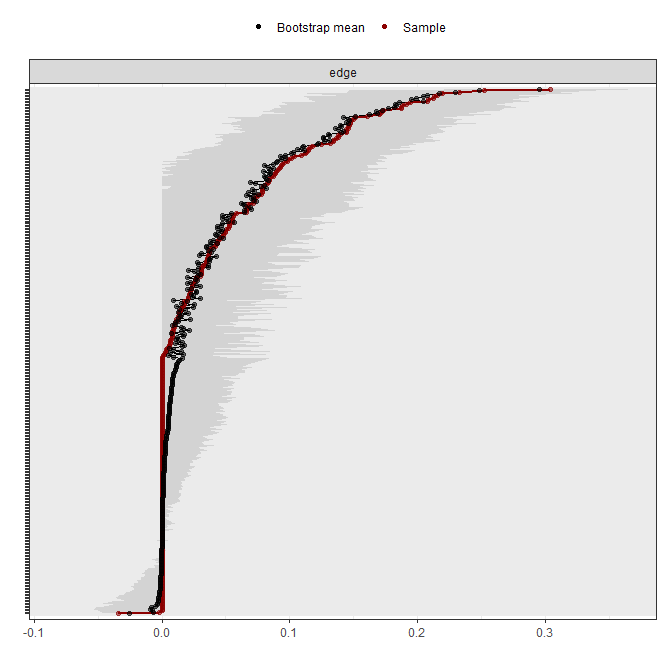


Figure S1. Nonparametric bootstrapped accuracy test for edge-weights of PDQ-D and GAD-7 items

The black dots indicate the initial edge weights, whereas the red line corresponds to the bootstrapped values. The grey area corresponds to confidence intervals (CIs), with narrower grey area indicate more accurate edge weights.


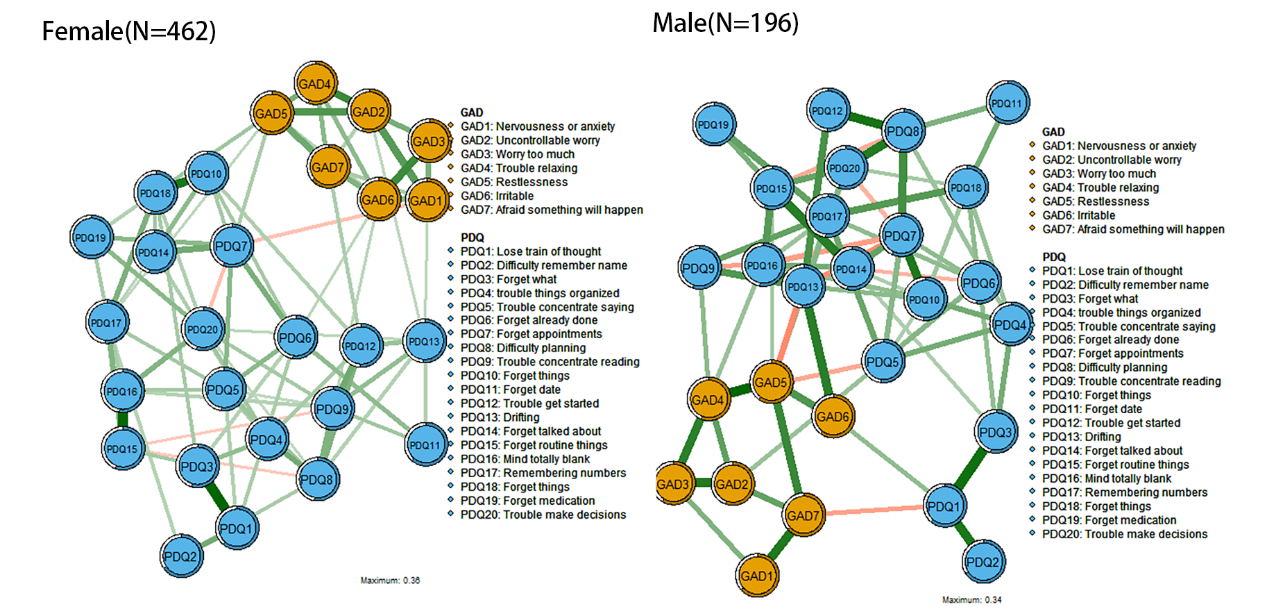


Figure S2. Estimated network models for anxiety and cognitive impairment symptoms in male and female participants

The different-size circles represent different strength of the nodes, while the width and saturation of edges indicate the connections and directions (i.e., green: positive correlation; red: negative correlation). The ring around each node indicates the predictability, with a filled ring representing that 100% of the variance is accounted for by the other nodes and an empty ring corresponding to 0% predictability. PDQ-D=Perceived Deficit Questionnaire for Depression, GAD=Generalized Anxiety Disorder.


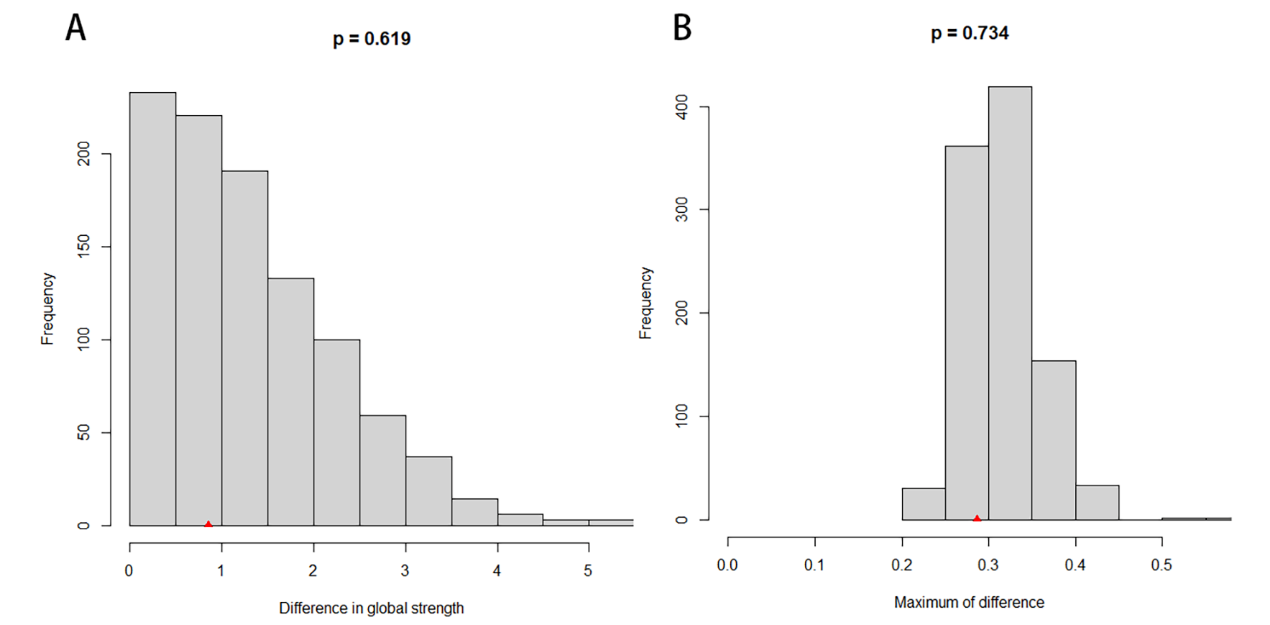


Figure S3. Statistical difference map of global strength and maximum of difference

The differences between the two networks (male vs female) were evaluated using the Network Comparison Test (NCT). Left Panel: Plot of bootstrap value of the maximum difference in any of the edge weights (1,000 permutations), with no significant difference (M=0.29, *p*=0.73). Right Panel: Plot of bootstrap value of the difference in network global strength, with significant difference (network strength among male participants: 11.82; among female participants: 12.67; S: 0.85, *p*=0.62). Invariance in edges weights was examined using the permutation test, generating sets of *p* values for each edge-edge comparison. Holm-Bonferroni corrected *p* values were all >0.05 indicating absence of significant differences.
